# Catatonia after COVID-19 infection: scoping review

**DOI:** 10.1192/bjb.2022.30

**Published:** 2023-08

**Authors:** Abdallah Samr Dawood, Ayia Dawood, Samr Dawood

**Affiliations:** 1 King's College London, UK; 2 University Hospital Lewisham, UK; 3 Oxleas NHS Foundation Trust, UK

**Keywords:** Catatonia, akinetic mutism, COVID-19, SARS-CoV-2, delirium

## Abstract

**Aims and method:**

Catatonia has been increasingly described in cases of COVID-19; we therefore aimed to investigate the evidence for catatonia in patients with COVID-19. We searched PubMed, EMBASE, PsycINFO, BIN and CINAHL databases for articles published in English, from the initial descriptions of the COVID-19 pandemic to January 2022.

**Results:**

A total 204 studies were identified, 27 (13%) of which met the inclusion criteria. The evidence available was based on case reports. The articles included in this review identified a total of 42 patients, ranging from the ages of 12 to ≥70 years, with confirmed or possible catatonia during or after a COVID-19 infection.

**Clinical implications:**

This review provides valuable information to clinicians in medical practice for treating patients with COVID-19, and a foundation for further research for this uncommon syndrome of COVID-19.

The speed and scale of the COVID-19 pandemic has been overwhelming and continues to rapidly spread, with various clinical presentations, including neuropsychiatric symptoms.^1^ Whether SARS-CoV-2 is neurovirulent or whether indirect generalised systemic changes exist behind these neuropsychiatric symptoms is not yet established.^2,3^ Recent studies have found structural changes to the brain, including various stages of gliosis, hypoxic changes, cerebral venous neutrophilic infiltrates and perivascular lymphocytic infiltrates, in addition to variable degrees of neuronal cell loss and axonal degeneration and injury.^2^

Data from observational studies on neuropsychiatric symptoms have recorded variability in the types and severity of the neurological and psychiatric manifestations of COVID-19.^1^ Although delirium was, expectedly, reported the most, other serious neuropsychiatric symptoms were reported too – namely, catatonia.^4^ Catatonia is well established as associated with serious physical complications, such as dehydration, respiratory aspiration, deep venous thrombosis or pulmonary emboli, and progression to malignant catatonia.^5^ Although untreated catatonia might have lethal outcomes, catatonia has a favourable treatment response. Catatonia is known to be underdiagnosed in non-psychiatric settings and is often misidentified as delirium, conversion disorder, psychosis or akinetic mutism, as many catatonic symptoms and signs are non-specific and are shared by various diagnostic possibilities.^6^ Subsequently, questioning the available evidence for the association between COVID-19 and catatonia is essential. Establishing a link between the two variables by analysing the available literature and data would raise important points and directions regarding treatment in patients with COVID-19. In this paper, we aim to identify the available findings regarding catatonia presentations in patients with COVID-19, and evaluate their connotations.

## Method

This study aims to review the current available literature on catatonia presentations of patients with COVID-19. We did a focused literature search of PubMed, EMBASE, PsycINFO, BIN and CINAHL databases. We used the following search parameters for each database: ‘((covid 19).ti,ab OR (COVID-19).ti,ab OR (SARS-CoV-2).ti,ab) AND ((stupor).ti,ab OR (catalepsy).ti,ab OR (waxy flexibility).ti,ab OR (mutism).ti,ab OR (mute).ti,ab OR (negativism).ti,ab OR (posturing).ti,ab OR (mannerisms).ti,ab OR (stereotypies).ti,ab OR (psychomotor agitation).ti,ab OR (grimacing).ti,ab OR (echolalia).ti,ab OR (echopraxia).ti,ab OR (Command automatism).ti,ab OR (automatism).ti,ab OR (catatonia).ti,ab)’. The search spanned from the initial descriptions of COVID-19 to 16 January 2022. The search criteria was designed to include each individual symptom of catatonia, to detect grey literature and potential catatonia cases that did not meet the full diagnostic criteria for catatonia and which might fall under the diagnosis of unspecified catatonia. Unspecified catatonia is an independent category in the DSM-5, which applies to presentations in which symptoms characteristic of catatonia cause clinically significant distress or impairment in social, occupational or other important areas of functioning, but either the nature of the underlying mental disorder or other medical condition is unclear, full criteria for catatonia are not met or there is insufficient information to make a more specific diagnosis. The creation of this category is aimed to allow for the rapid diagnosis and specific treatment of catatonia in severely ill patients. Of note, in the DSM-IV, two out of five symptom clusters were required to diagnose catatonia if the context was a psychotic or mood disorder, whereas only one symptom cluster was needed if the context was a general medical condition.

We included any study (case report, case series and observational studies) written in English which explored and reported the presence of catatonia as a syndrome, or the co-occurrence of a minimum of two symptoms of catatonia, particularly the two core symptoms of stupor and mutism, in patients with COVID-19. Articles were excluded if they reported catatonic symptoms in the context of a secondary reaction to the psychological stresses associated with the COVID-19 pandemic, or because of the psychological implications of public lockdown. Animal studies and studies in languages other than English were also excluded. This review followed the PICO (population, intervention, control and outcomes) structure and technique to frame the search as a diagnostic problem. Level of evidence were rated according to the Oxford Centre for Evidence-Based Medicine (https://www.cebm.net/contact/), a UK-based service providing rapid evidence reviews and data analysis relating to the coronavirus pandemic, which is affiliated with Oxford University's Nuffield Department of Primary Care Health Sciences.

This paper did not collect data from patients or patients’ clinical notes, hence there was no need to obtain ethical approval.

## Results

Between the start of the COVID-19 pandemic in 2019 and January 2022, we identified a total of 204 studies (71 [35%] from PubMed, 90 [44%] from EMBASE, 22 [11%] from PsycINFO, four [2%] from BIN and 17 [8%] from CINAHL). Considering the rarity of catatonia diagnoses, the total number of studies obtained was high. This finding is possibly a result of our search parameters, which included identifying and including studies that discussed the co-occurrence of individual symptoms that form the diagnostic criteria of catatonia. Of the 204 studies identified, 27 (13%) met our inclusion criteria. Table 1 summarises the 27 studies included in this review.

**Table 1:** Included publications and brief notes from their findings

Author	Type of study	Patient/sample	Diagnostic test	Level of evidence	Past medical and psychiatric history	Short clinical notes
Blum et al^7^	Case report	A man in his 60 s	BFCRS	4	History of hypertension and psychotic episode (20 years ago)	Patient presented to the emergency department with 3 days of altered mental state: muteness, staring, perseveration, ambitendency, rigidity, gegenhalten agitation, verbigeration, insomnia and refusal of food and water. Fifteen minutes following a lorazepam challenge, BFCRS score decreased from 27 to 9
Kopishinskaia et al^8^	Case series	A 12-year-old male	Clinical examination	4	History of perinatal encephalopathy, brief febrile seizures at 6 months; nil previous psychiatric history	Summer 2020: showed slowness in movement and weight loss owing to reduced appetite and spontaneous fluid intake. Admitted on 10 December 2020 because of neurological sequalae of COVID-19 inflammatory diseases. During admission, the patient demonstrated the pillow sign within catatonic rigidity, posturing and slow gait with festination gait
		A 17-year-old male	Clinical examination	4	Nil previous physical health history; nil previous psychiatric history	May 2020: patient experienced psychiatric and neurological symptoms. January 2021: admitted to a psychiatric hospital, where he was treated with risperidone. No psychiatric diagnosis was conveyed, but he tested positive for IgM antibodies to SARS-CoV-2. 26 January 2021: the patient was sitting in a hunched posture, showed understanding of the conversation and replied appropriately to questions, but answered rapidly and abruptly, in telegraphic speech
Tyler Torrico et al^9^	Case series	A 36-year-old Black female	Clinical examination	4	Past medical history of hypertension, type 1 diabetes and incidental pancreatic head mass; nil previous psychiatric history	Patient presented with multiple symptoms of catatonia 26 days after developing symptoms of COVID-19. Initial treatment with methylprednisolone resulted in no improvement. Catatonia was suspected and 2 mg lorazepam trial was initiated. Within 10 min after the first dose of lorazepam, the patient sat up at the bedside and was seen calling her family members
		A 64-year-old White female	Clinical examination	4	Nil previous medical history; past psychiatric history of unspecified bipolar disorder	Two months of symptoms of waxing and waning mentation problems, selective mutism, minimal engagement with others and staring. Refusal to eat or drink, resulting in 20-pound weight loss. COVID-19 RNA was detected by RT-PCR 6 weeks before presentation. The patient was given 2 mg intravenous lorazepam for suspected seizure. Shortly thereafter, she was entirely alert and oriented. Her physical health deteriorated and was treated with methylprednisolone, with no benefit. A lorazepam trial was re-initiated, starting at 0.5 mg lorazepam three times daily. Over the next 3 days, the patient resumed speech
Mulder et al^10^	A case report	A 40-year-old male	Clinical examination	4	Nil previous physical health history; nil previous psychiatric health history	Patient presented with multiple sever catatonic symptoms after 3 weeks history of COVID-19; no hospital admission needed. Signs of autoimmune encephalitis. Treatment was initiated with 1 g methylprednisolone daily. On day 28, the patient showed dramatic improvement
Kwon et al^11^	A case report	A 62-year-old woman	BFCRS	4	Past medical history of hypertension; past psychiatric history of schizoaffective disorder and bipolar disorder	Patient presented to the emergency department with shortness of breath and weakness for 1 week; a COVID-19 test was positive. On hospital days 6–9, the patient displayed extreme mutism and negativism. On hospital day 10, a single 1 mg trial of lorazepam was administered for a presumed diagnosis of catatonia with no improvement. On hospital day 18, patient was started on intravenous lorazepam every 8 h. After 48 h of starting the lorazepam challenge, the patient had a significant improvement. Patient died because of sudden cardiac arrest refractory to resuscitation efforts; likely secondary to a massive pulmonary embolism
Vazquez-Guevara et al^12^	A case report	A 43-year-old female	BFCRS	4	Nil previous physical health history; nil previous psychiatric health history	Patient presented with 3-day history of cationic symptoms. BFCRS: 19 points. Diazepam infusion for the catatonic syndrome was started, with no good response. Serum IgG, IgM and PCR were positive for COVID-19. Methylprednisolone for 5 days was given for encephalitis because of a lack of diagnostic certainty, followed by oral prednisone. Remarkable improvement was shown with the boluses of steroids
Scheiner et al^13^	Case series and systemic review	A female in her 50 s	BFCRS	4	History of hypertension and osteoarthritis; nil previous psychiatric history	Patient presented with self-inflicted stab wounds, catatonic features and positive SARS-CoV-2 nasopharyngeal swab. BFCRS score of 11. She was challenged with 2 mg intravenous lorazepam, with lysis of catatonia (BFCRS severity 0) after ~45 minutes
		A female in her 50 s	BFCRS	4	History of chronic kidney disease stage 3; history of chronic schizophrenia	Patient brought to the emergency department from a local jail twice for refusal to eat; 50-pound weight loss over 2 months. SARS-CoV-2 swab was positive. BFCRS severity of 12. Symptoms included withdrawal, mutism, staring and negativism. Patient was administered a 2 mg intramuscular lorazepam challenge, with good response (BFCRS severity 2)
Zain et al^14^	A case report	A 69-year-old female	Clinical examination	4	Past medical history of chronic obstructive pulmonary disease, type 2 diabetes and hypertension; nil previous psychiatric history	Patient presented with confusion and paranoid thoughts for 4–6 weeks, worsening over the past 2 weeks. PCR test for COVID-19 at the time of admission was negative. Antibody test was done on day 6 of admission, which was positive. She displayed extreme agitation and aggression, poor eye contact and had a flat affect with echolalia and loose associations. Excited catatonia was considered as a possible delayed catatonic reaction to COVID-19 after 2 months, with differential diagnosis of delirium and dementia. She was treated with lorazepam; agitation and paranoia appeared to improve
Jaber et al^15^	Report of two patients	A 39-year-old male	DSM-5 diagnostic criteria	4	Nil previous physical health history; nil previous psychiatric history	Patient presented with delirium and catatonic features. Admitted to the ICU. MRI and CSF were normal, but the patient tested positive for SARS-CoV-2 and was treated successfully with lorazepam
		A 56-year-old female	DSM-5 diagnostic criteria	4	Nil previous physical health history; history of schizoaffective disorder	Patient presented with delirium, and catatonic features. Admitted to the ICU. MRI and CSF were normal, but the patient tested positive for SARS-CoV-2 and was treated successfully with lorazepam
Jaber et al^16^	A case report	A 36-year-old female	Clinical examination	4	History of diabetes; nil previous psychiatric history	Patient presented with slurred speech, which progressed to catatonia, and tested positive for COVID-19. The patient received intravenous methylprednisone out of concern for autoimmune or paraneoplastic encephalitis; showed no clinical improvement. Symptoms quickly resolved in response to lorazepam
Johnson et al^17^	A case report	A 27-year-old male	Clinical examination	4	A history of benign pressure hydrocephalus and polycythaemia; nil previous psychiatric history	Patient presented with depression and catatonia, and was transferred to a tertiary care centre for ECT. Upon admission, patient was found to be COVID-negative but tested positive on day 40 of the admission, after exposure to a COVID-19-positive visitor, and catatonia progressed to malignant catatonia. Patient initially improved with scheduled lorazepam and ECT, with partial regain in motor function. However, overnight on hospital day 40, patient acutely spiked fevers to 40 degrees Celsius, had acute worsening in rigidity and rapidly rising CPK level from 166 to 3200 in just over 48 h
Fiaschè et al^18^	A case report	A 59-year-old male	Clinical examination	4	Nil previous physical health history; a history of schizotypal personality disorder	Patient presented with acute symptoms of COVID-19 and catatonic behaviour manifested by stuporous state, catalepsy, immobility, rigidity and mutism. Treated with successfully with asenapine 20 mg/day; symptoms responded rapidly and significantly
Nersesjan et al^19^	A prospective, consecutive, observational study	61 patients aged ≥18 years, consecutively admitted with COVID-19 to a tertiary referral centre in Copenhagen	No test used; the diagnosis was based on the clinical pictures. Other scales were used to test the level of consciousness: Glasgow Coma Scale and Full-Outline UnResponsiveness (FOUR) scale	1b		Out of the 61 patients included, a total of 19 patients had signs of encephalopathy. Eight patients were apathetic and profoundly hypokinetic, with almost no voluntary movements and without verbal output. Patients were able to indicate yes and no by nodding with their heads when spoken to. On neurological examination, all patients had some frontal release signs (e.g. palmomental reflexes). They were diagnosed with akinetic mutism
Zandifar and Badrfam^20^	A case report	A 61-year-old male	Clinical examination	4	Nil previous physical health history; a history of schizophrenia for many years	Worsening of schizophrenia and sudden onset of seizure. PCR test for COVID-19 was positive. Patient developed catatonic symptoms and was treated with lorazepam 2 mg three times a day. Within 24 h, his catatonic symptoms resolved. After 36 h, his lethargy decreased, and he started consuming water and food
Deocleciano de Araujo et al^21^	A case report	A 50-year-old male	Clinical examination	4	Nil previous medical history; history of mild intellectual disability	Symptoms started a week before admission; severe body stiffness, negativism, urinating and defecating in clothes, and refusal to feed and weight loss, which required ICU admission and ECT. A COVID-19 test was positive. Oral lorazepam 2 mg three times daily was given. Despite benzodiazepines, stiffness and diaphoresis remained extreme, and he remained in critical condition. On day 19, he was transferred from intensive care to the psychiatric unit. ECT with bilateral stimulus 30% was started. After ten ECT sessions, the catatonic syndrome improved substantially. However, he unable to recall the time during admission and the weeks prior
Gouse et al^22^	A case report	An elderly male	BFCRS	4	History of chronic obstructive pulmonary disease, interstitial lung disease, type 2 diabetes, hypertension, atrial fibrillation and essential tremor; history of schizophrenia	Patient presented to the emergency department with worsening COVID-19 symptoms. On hospital day 3, he exhibited catatonia. Lorazepam 1 mg was initiated intravenously. On reassessment 30 min post dose, his BFCRS improved from 18 to 9. Because of concern for catatonia, his aripiprazole was held and intravenous lorazepam 1 mg three times daily was initiated. On day 4, his BFCRS score was 13. Intravenous lorazepam was decreased to 0.5 mg three times daily, by his primary team, because of concern for his progressive respiratory failure; patient had a do-not-intubate status. On day 5, his BFCRS was 19. An ECT consult was placed but declined, because of concern for his medical stability. The patient died on day 7.
Amouri et al^23^	A case report	A 70-year-old female	BFCRS	4	Medical history of diabetes mellitus type 2, end-stage renal disease on haemodialysis, hypertension, coronary artery disease, hypothyroidism and a history of transient ischemic attack; nil previous psychiatric history	Patient presented to the hospital with a few days history of physical and psychiatric symptoms and recent positive test result for COVID-19. On day 12, the patient had a BFCRS of 11/21, indicating the presence of catatonia and delirium. She received eight total doses of lorazepam 0.5 mg between day 12 and 15. On day 15, she showed improved alertness and orientation. Mild symptoms of catatonia remained stable. Lorazepam was subsequently discontinued on day 16, without the return of catatonic signs
Pilotto et al^24^	A case report	A 60-year-old male	Clinical examination	4	Nil previous physical health history; nil previous psychiatric history	Patient presented to the emergency department with a severe alteration of consciousness. On admission, the patient showed a severe akinetic syndrome associated to mutism. He was diagnosed with COVID-19-induced encephalitis. Three days after admission, intravenous steroid treatment was started (methylprednisolone 1 g/day for 5 days), with a good response
Caan et al^4^	A case report	A 43-year-old male	Clinical examination	4	Nil previous physical health history; nil previous psychiatric history	Developed catatonic features 2 weeks post positive test of COVID-19; classic symptoms of catatonia. He responded immediately to lorazepam
Huarcaya-Victoria et al^25^	A case report	A 23-year-old female	Clinical examination	4	Nil previous physical health history; nil previous psychiatric history	Patient was admitted with COVID-19 symptoms, agitation and delusion, and the following catatonic symptoms: stereotyped movements, catalepsy, verbigeration. Patient was given intravenous midazolam to treat the agitation, with little effect. Treated successfully with 40 mg ziprasidone followed by olanzapine, with a good response
Raidurg et al^26^	A case report	A 28-year-old female	BFCRS	4	Nil previous physical health history; nil previous psychiatric history	History of 3 weeks psychotic behaviour. After admission to a private psychiatry hospital, she stopped talking completely. Her BFCRS score was 16. A lorazepam challenge test was done, to which her catatonic features responded well. Patient was discharged on day 8 with 90% remission, on tablet lorazepam 1 mg twice daily, and on tablet olanzapine 5 mg
Palomar-Ciria et al^27^	A case report	A 65-year-old male	Clinical examination	4	Hypertension and sleep apnoea syndrome treated with CPAP; schizophrenia for more than 20 years	Patient presented with bizarre behaviour for 20 days, with positive results for both IgG and IgM, but PCR was then negative. He was diagnosed with delirium owing to COVID-19 with a relapse in his schizophrenic illness, and discharged from the hospital. He was taken to the emergency room after a few days, with suicidal behaviour. During the second hospital admission, the predominant symptoms were disorganised behaviour, reiterative or blocked speech, bradyphrenia, disorientation, apraxia, echolalia and bradykinesia without rigidity. He was diagnosed with catatonia and treated with ECT
Austgen et al^28^	A case report	A 52-year-old female	BFCRS	4	History of well-controlled type 2 diabetes, mild hypertension and post-viral reactive airway disease periodically requiring oral steroids; nil previous psychiatric history	A month after testing positive for COVID-19 by PCR, she was admitted to hospital because of depression and paranoia. She developed the following symptoms of catatonia: immobility, negativistic and mutism, with a BFCRS score of 18. She was treated with ECT and concurrent use of lorazepam 8 mg daily, and she improved
Kaur et al^29^	A case report	A 59-year-old male	BFCRS	4	Past medical history included well-controlled hypertension and hyperlipidaemia; a history of bipolar spectrum illness	Patient presented with severe depression; PCR COVID-19 testing was negative on admission. He received Moderna two-dose COVID-19 vaccination series approximately 4 months before admission. Within 7 days of admission there was an abrupt onset of catatonia. Repeat PCR testing was performed and was positive for COVID-19. Oral lorazepam was started and titrated. He was unable to tolerate lorazepam beyond 6 mg daily and titration was stopped. He received six brief-pulse ECT bitemporal treatments, with excellent response
Flannery et al^30^	A case report	A female in her 20s	Clinical examination	4	Nil previous physical health history; nil previous psychiatric history	Constellation of symptoms (spontaneous defecation, catatonia, sudden encephalopathy without metabolic or infectious findings) coupled with preliminary CSF results and history of deterioration after SARS-CoV-2 vaccination led to a strong clinical suspicion of an autoimmune-mediated encephalitis driven by the vaccine. Catatonia appeared after more than 2 weeks, and was treated with rituximab, antipsychotics and steroids
McHattie et al^31^	A case report	53-year-old female	Clinical examination	4	Ductal carcinoma of breast in remission and psoriasis; history of depression	Two-week history of confusion and catatonia. Her swab for SARS-CoV-2 RNA was negative and was treated with acyclovir for suspected viral encephalitis. She tested positive for COVID-19 on day 14. Treatment included hydroxychloroquine, intravenous immunoglobins, tocilizumab, antibiotics, amphotericin and levetiracetam. After 1 month, she made remarkable progress
Sakhardan et al^32^	Case series	A 21-year-old male	BFCRS	4	Underlying genetic condition, early-onset Parkinson's disease; nil previous psychiatric history	A month's history of mutism, withdrawal, staring, posturing, urinary incontinence, autonomic instability, immobility, rigidity, negativism, waxy flexibility and postural instability, with symptomatic worsening over the preceding 4 days. The patient was initiated on oral lorazepam but switched to intravenous lorazepam (6 mg daily) in three divided doses, because of inadequate response (BFCRS score 17). Bitemporal ECT, administered thrice weekly, were commenced following non-response to lorazepam (BFCRS score 9). Improvement after the tenth ECT session was observed. Given persistent rigidity and gait instability, a neurology opinion was obtained and a diagnosis of atypical Parkinson's disease was considered.
		A 26-year-old male	BFCRS	4	Nil previous physical health history; history of schizophrenia	One month's history of stupor, mutism, staring, posturing and grimacing suggestive of catatonia. There was no prior history of catatonia. Catatonic signs improved with the lorazepam challenge (2 mg intravenous), in-patient care was planned for the management of residual catatonia and psychotic illness. Oral lorazepam (6 mg daily in divided doses) resulted in the resolution of catatonia over 2 days (BFCRS score 0)
		A 38-year-old female	BFCRS		Nil previous physical health history; nil previous psychiatric history	Patient presented with 4-day history of psychotic symptoms plus mutism, posturing, staring, withdrawal, immobility and automatic obedience, in the absence of any prior history of catatonia or psychiatric illness. COVID-19 exposure was present (immediate family member). The patient was diagnosed with acute transient psychotic disorder with catatonia (baseline BFCRS score of 8). RT-PCR test was positive. No respiratory or systemic abnormalities were noted. She was admitted to the PCU and commenced on oral olanzapine 10 mg daily, and lorazepam 4 mg daily (divided doses). Catatonia resolved over a week (BFCRS score 0)
		A 24-year-old male	BFCRS		Nil prior physical health history; history of schizoaffective disorder	Patient presented with 2 weeks of immobility, mutism, staring, posturing, grimacing, mannerism, negativism, withdrawal, impulsivity and ambitendency. Intravenous lorazepam (8 mg in divided doses) was associated with improvement in food intake. Subsequently, it was replaced with oral lorazepam (8 mg daily) and oral aripiprazole titrated to 20 mg. As inadequate response to lorazepam was noted, bifrontal ECT were commenced. Following the first ECT, he was identified as COVID-19 positive and. given the persisting residual mutism, staring, grimacing and impulsivity, ECTs were commenced. A course of five bifrontal ECT sessions contributed to improvement in catatonia and mood symptoms. He was discharged on lorazepam 2 mg. On follow-up after 2 weeks, he was readmitted for worsening catatonia with immobility, mutism, staring, grimacing and ambitendency (BFCRS score of 7). Oral lorazepam up to 6 mg and nine bifrontal ECT sessions were administered, which improved both catatonic (BFCRS score 0) and psychiatric symptoms
		A 35-year-old female	BFCRS		History of hypothyroidism; history of schizoaffective disorder	No history of catatonia was noted in the past, but she had received ECT for psychiatric relapses. Patient presented with a 1-month duration of depressive symptoms and 4 days of stupor, negativism, mutism, posturing, staring, gegenhalten and withdrawal (BFCRS score 18). In the emergency ward, catatonia responded to intravenous lorazepam (6 mg daily); however, negativism and withdrawal persisted, for which in-patient care was planned. The RT-PCR test was positive, requiring admission to PCU. A concurrent course of 7 bitemporal ECT sessions (twice weekly, with first three ECT sessions in the PCU with precautions) contributed to gradual improvement in catatonic (BFCRS score 0) and depressive symptoms. Parenteral lorazepam was switched to oral lorazepam 6 mg daily and gradually tapered and stopped over 2 weeks

BFCRS, Bush–Francis Catatonia Rating Scale; IgM, immunoglobulin M; RT-PCR, reverse transcription polymerase chain reaction; IgG, immunoglobulin G; PCR, polymerase chain reaction; ICU, intensive care unit; MRI, magnetic resonance imaging; CSF, cerebrospinal fluid; ECT, electroconvulsive therapy; CPK, creatine phosphokinase ; CPAP, continuous positive airway pressure; PCU, progressive care unit.

Application of the inclusion criteria was based on two stages of screening: first, the titles and the abstracts, followed by the full texts of those studies to identify description of catatonia. Twenty-five (12%) of 204 articles recorded a diagnosis of catatonia as a comorbidity to COVID-19, and all were either an individual case report or a series of case reports. One (<1%) study reported the diagnosis of akinetic mutism in eight patients, but they exhibited the two core symptoms of catatonia, and one (<1%) study reported catatonic features in a patient with encephalitis and delirium. Both studies were included as they recorded mutism and a hypokinetic state in their patients, indicating the possibility of unspecified catatonia.

The 27 included articles in this review observed a total of 42 patients, ranging from the ages of 12 to ≥70 years. Of those patients, 17 (40%) were female and 17 (40%) were male, with no demographic data available for eight (10%) patients. The largest cluster of patients were aged >50 years (17 [40%] out of 42 patients); among them, nine (53%) out of 17 patients were aged >60 years. With the increase in the prevalence of COVID-19 in individuals aged <18 years, reports concerning catatonia in COVID-19 in younger individuals have begun to appear. Two (5%) out of 42 patients identified in this review were aged <18 years. The Bush–Francis Catatonia Rating Scale was used as a diagnostic tool to identify catatonia in ten (24%) out of 42 patients, and 32 (76%) patients were identified depending on their clinical examination.

Age, previous history of certain physical health problems and previous psychiatric history are predisposing factors for catatonia in COVID-19. The psychiatric premorbid diagnoses reported in these cases included bipolar disorder in two (5%) patients, schizophrenia in five (12%) patients, schizoaffective disorder in four (10%) patients, schizotypal personality disorder in one (2%) patient, depression in one (2%) patient and mild intellectual disability in one (2%) patient. The most common physical premorbid diagnoses were hypertension, followed by diabetes, chronic kidney diseases, chronic lung diseases, ischaemic vascular diseases, neurological diseases and hypothyroidism, which were all reported separately or in combination. Ductal carcinoma of the breast in remission and psoriasis were also reported in one patient who presented with encephalitis and catatonia. No previous psychiatric or physical health problems were reported in ten (24%) out of 42 patients (Table 1).

## Discussion

Taking in consideration the number of cases identified, the outcome of this review has presented catatonia as a possible complication of COVID-19, regardless of age, gender and the background of the physical or mental health status of patients. However, this review indicates that the strength of evidence for catatonia in COVID-19 was poor, as there are no robust observational studies available. The below discussion represents the 34 patients whose data were available. The presentation of catatonia in COVID-19 varied in onset, severity and duration. Catatonic states were reported throughout the acute and subacute phases of COVID-19, as well as a long-term post-encephalitis neuropsychiatric complication. Catatonia occurred in combination or independently of any other psychiatric symptoms. However, catatonia mainly arose concurrent with the acute phase of COVID-19, but was reported as a long-term complication for COVID-19 (Table 1).

Lorazepam was used in 22 patients, and demonstrated notable and even rapid improvements in the core symptoms of catatonia, including mutism, echolalia, rigidity, waxy flexibility and automatic obedience in 18 patients; the remaining four patients needed further electroconvulsive therapy (ECT).^21,29,32^ Furthermore, lorazepam led to mild improvements in delirium in one patient,^23^ and improvement in agitation and paranoia in another.^14^ Oral or intramuscular lorazepam challenge tests led to lysis of catatonia after 10–30 min in most cases. For example, a report from the Kern Medical Centre in California described two cases of suspected catatonia associated with COVID-19 who responded immediately to lorazepam injections, with marked improvement in their symptom profile.^9^ Delaying treatment with benzodiazepines because of worries about worsening delirium might be followed by serious physical complications, such as pulmonary embolism.^11^ Steroid therapies were trialled in seven patients that were diagnosed with COVID-19-induced encephalopathy, which proved useful in four (57%) patients.^9,10,12,16,24,28^ In the remaining three (43%) patients, steroid therapy failure was followed by successful lorazepam treatment.^9,16,28^ This finding suggests that the well-known efficacious responses to lorazepam therapy in catatonia is still valid in COVID-19, with almost a complete resolution of all catatonic signs in most cases. No serious unwanted side-effects were recorded. Although lorazepam is helpful in treating catatonia, lorazepam alone might not suffice. ECT should always have serious early consideration in catatonia, as it showed good results and safety even with the physical health complications of COVID-19.^17,21,27–29,32^ In one patient, nonetheless, ECT was declined because of concerns regarding physical health problems.^22^ Despite the controversy surrounding antipsychotics in catatonia, two case reports described efficacious treatments utilising the antipsychotics olanzapine, asenapine and ziprasidone.^12,25^

In another aspect, COVID-19 has the potential to negatively affect the clinical course of established catatonia, as highlighted by the study by Johnson et al^17^ which described a 27-year-old male with a past medical history of normal pressure hydrocephalous and polycythaemia. He was first admitted for depression and increasing catatonic symptoms. He tested negative for COVID-19 on admission, but was infected during in-patient stay, and his catatonia deteriorated to malignant catatonia. The COVID-19 vaccinewas also reported as a possible cause for the catatonia presentation and it was treated with rituximab, an antipsychotic and steroids.^30^

Nersesjan et al^19^ enrolled 61 patients with COVID-19 who were admitted to Rigshospitalet, Copenhagen University Hospital, a tertiary referral centre in Denmark; the group identified encephalopathy in 19 patients, eight (42%) of whom were apathetic and severely hypokinetic or akinetic, with almost no voluntary movements and no verbal output. However, these eight patients were conscious and appeared aware of their surroundings through intelligible gaze responses. Those patients were given the diagnosis of akinetic mutism, and these symptoms lasted for a median of 4 days. These clinical observations could raise the possibility of unspecified catatonia. The Guidelines and Evidence-Based Medicine Subcommittee of the Academy of Psychosomatic Medicine states that the disorder fulfils DSM-5 criteria for ‘catatonic disorder due to another medical condition’, as all cases of akinetic mutism involve stupor, immobility and mutism,^33^ However, the guidelines recognise that this definition is complicated by the fact that some medical conditions can cause both akinetic mutism and catatonia.^33^

Additionally, Pilotto et al^24^ described a case of a 60-year-old patient with severe acute respiratory syndrome owing to COVID-19, who had only mild respiratory abnormalities and developed an akinetic mutism with abulia. He was considered as suffering from encephalitis and delirium secondary to COVID-19; however, this case report's clinical description fulfilled at least two of the diagnostic criteria of catatonia. Additionally, 3 days after admission, given the persistence of clinical symptoms, high-dose intravenous steroid treatment was started (methylprednisolone 1 g per day for 5 days) and was effective. In an exploratory study, Grover et al^34^ systematically demonstrated that 30.2% of patients with delirium, regardless of aetiology, met the criteria for catatonia by scoring positive on two of the first 14 items of the Bush–Francis Catatonia Rating Scale. These findings suggest that catatonia can coexist with delirium.^33,34^ Following Grover et al's report, several successive case reports were published describing encephalitis and delirium with catatonia in the context of COVID-19 infection.^10,12,15,29^ Lorazepam challenge test is widely used to either confirm or exclude catatonia.^35^ In such ambiguous presentations, a challenge test with lorazepam would be beneficial.

Catatonia shares symptoms with a wide spectrum of physical and mental illnesses, such as status epilepticus, akinetic mutism, delirium, metabolic conditions (e.g. diabetic ketoacidosis), neuroleptic malignant syndrome, Parkinson's disease, extrapyramidal side-effects, substance misuse disorders, depressive disorders and psychosis. These examples are common illnesses that could be part of the aetiology for the progression and presentation of catatonia.^36^ Many of these conditions were described in patients with COVID-19 identified in our review.^37–40^ Consequently, misclassification or missed diagnoses of catatonia is a possibility, and many cases fall within the grey zones of disease classification. Grey zones in medicine represent a complex situation and a great challenge to the diagnostic classification and subsequent treatment of patients.^41^

In conclusion, our review was limited by the paucity of cohort studies and case–control studies designed to evaluate the epidemiology of catatonia and its course in COVID-19. The diagnostic problem was explored mainly through case reports, and the majority did not use a diagnostic rating scale. The diagnostic uncertainty extends to the cohort study included in this review that reported akinetic mutism in patients with COVID-19, which did not use diagnostic methods to examine the differential diagnosis and determine a precise and exclusive diagnosis.

Nevertheless, according to the reports available, evaluation of the possibility of catatonia in patients with COVID-19 infection and those presenting with mutism and hypokinesia or other symptoms of catatonia is important. As the pandemic continues, increasing the awareness of the diagnosis of catatonia among medical staff who are working with patients with COVID-19 would facilitate a timely identification and management of catatonia, which will have significant and lifesaving outcomes on the acute treatment of these cases.

Most patients with catatonia responded substantially and rapidly to benzodiazepines. The role of antipsychotic agents in treatment is controversial. ECT was effective, but would present a challenging circumstance in the prevention and treatment of medical complications of COVID-19.

## Data Availability

The data that support the findings of this study are available on request from the corresponding author, S.D., upon reasonable request. The searching protocol used in this review is available within the study.

## About the authors

**Abdallah Samr Dawood** is a medical student at the Department of Medicine, GKT School of Medical Education, King's College London, UK. **Ayia Dawood** is a STEP pharmacist with the Pharmacy department at University Hospital Lewisham, Lewisham and Greenwich NHS Trust, UK. **Samr Dawood** is a consultant psychiatrist with the Department of Medical Education, Oxleas NHS Foundation Trust, UK.
